# The Kinetics of G2 and M Transitions Regulated by B Cyclins

**DOI:** 10.1371/journal.pone.0080861

**Published:** 2013-12-04

**Authors:** Yehong Huang, R. Michael Sramkoski, James W. Jacobberger

**Affiliations:** 1 Department of Molecular Biology and Microbiology, Case Western Reserve University, Cleveland, Ohio, United States of America; 2 Case Comprehensive Cancer Center, Case Western Reserve University, Cleveland, Ohio, United States of America; Emory University, United States of America

## Abstract

B cyclins regulate G2-M transition. Because human somatic cells continue to cycle after reduction of cyclin B1 (cycB1) or cyclin B2 (cycB2) by RNA interference (RNAi), and because cycB2 knockout mice are viable, the existence of two genes should be an optimization. To explore this idea, we generated HeLa BD™ Tet-Off cell lines with inducible cyclin B1- or B2-EGFP that were RNAi resistant. Cultures were treated with RNAi and/or doxycycline (Dox) and bromodeoxyuridine. We measured G2 and M transit times and 4C cell accumulation. In the absence of ectopic B cyclin expression, knockdown (kd) of either cyclin increased G2 transit. M transit was increased by cycB1 kd but decreased by cycB2 depletion. This novel difference was further supported by time-lapse microscopy. This suggests that cycB2 tunes mitotic timing, and we speculate that this is through regulation of a Golgi checkpoint. In the presence of endogenous cyclins, expression of active B cyclin-EGFPs did not affect G2 or M phase times. As previously shown, B cyclin co-depletion induced G2 arrest. Expression of either B cyclin-EGFP completely rescued knockdown of the respective endogenous cyclin in single kd experiments, and either cyclin-EGFP completely rescued endogenous cyclin co-depletion. Most of the rescue occurred at relatively low levels of exogenous cyclin expression. Therefore, cycB1 and cycB2 are interchangeable for ability to promote G2 and M transition in this experimental setting. Cyclin B1 is thought to be required for the mammalian somatic cell cycle, while cyclin B2 is thought to be dispensable. However, residual levels of cyclin B1 or cyclin B2 in double knockdown experiments are not sufficient to promote successful mitosis, yet residual levels are sufficient to promote mitosis in the presence of the dispensible cyclin B2. We discuss a simple model that would explain most data if cyclin B1 is necessary.

## Introduction

Eukaryotic cell cycle progression is regulated by cyclin-dependent kinases (Cdks) and their regulatory, cyclin subunits [1]–[4]. Cdk cell cycle expression is proportional to cell mass in excess of cyclins, which are limiting and expressed periodically. This periodicity, in part, creates periods of activity for specific cyclin complexes that correlate roughly with cell cycle phases and/or major cell cycle events [5]. Assignment of cyclin/Cdk activity to major cell cycle events has been studied in most model organisms, and cyclin/Cdk complexes activate transcription [6], [7], enable DNA replication [8], [9], and catalyze mitosis [5].

Cdc2 or cyclin-dependent kinase 1 (Cdk1) regulates mitotic entry and progression [10]. Expression of a kinase-dead mutant or immunodepletion causes G2 arrest in human cells [11], [12]. Conditional, down-regulation of Cdk1 stops HT2-19 human cell division and promotes endoreduplication [13]. Chemical inhibition of Cdk1 arrests interphase cells in G2, but in mitotic cells, results in premature origin licensing and mitotic exit [14]. In mitosis, A and B type cyclins activate Cdk1. Cyclin A is required for G2 to M transition and nuclear envelope breakdown [15], [16], however B cyclins are the principal activators of Cdk1. Cyclin B-Cdk1 complexes are activated by a cdc25 phosphatase [17]. The activated complex then phosphorylates a large number of substrates to regulate sub-cellular events, including mitotic entry, chromosome condensation, nuclear envelope breakdown, spindle assembly, Golgi fragmentation, and the spindle checkpoint (reviewed in [10]). The complex is inactivated at the metaphase to anaphase transition when B cyclins are degraded by the anaphase promoting complex/cyclo some (APC/C) [18]. In mammals, there are three B cyclins: B1, B2 and B3. Cyclin B3 is expressed in the human testis and in developing germ cells in the mouse [19], [20]. Cyclin B1 and B2 differ in the first 100 residues, and are 57% identical in the remaining sequences [21], [22]. Mammalian cyclins B1 and B2 are co-expressed. They are detectable beginning in G1, rise slowly through S phase then rapidly in G2, peaking in late G2 or early M, and degraded approximately after metaphase [23]–[26]. Cyclin B1 shuttles between the cytoplasm and nucleus but is mostly cytoplasmic during interphase and mostly nuclear in prophase with initial activation on the centrosome [24], [27]–[29]. Cyclin B2 localizes to the Golgi apparatus and evidence supports a role in regulating Golgi fragmentation [24], [30]–[35].

Different localization suggests different roles for cyclin B1 and cyclin B2, and exogenous expression in G1 cells coupled with amino termini swapping demonstrated that cyclin B1 regulated mitotic events like cell rounding, chromatin condensation, aster formation, and nuclear membrane breakdown while cyclin B2 regulated Golgi fragmentation. However, cyclin B1 with a B2 amino terminus was capable of Golgi fragmentation while cyclin B2 with amino terminal B1 residues was not capable of nuclear mitotic functions despite apparently correct cytoplasmic localization [31]. Since these exogenous proteins were all expressed at about the same levels, the experiments suggested that localization may have a significant effect on substrate specificity, but the termini swapping also suggested substrate differences between the two cyclins that are not dependent on localization. However, experiments with B cyclin Null mice have shown that cyclin B1 is required for the viability of embryos, but cyclin B2 is not [21]. Thus, mammalian B cyclins appear to have distinctly different functions; different substrates are implied, but cyclin B2 function is dispensable.

RNA interference (siRNA) experiments for both cyclins B1 and B2 in human somatic cell lines results in G2 arrest while knockdown of either cyclin B1 or cyclin B2 alone results in mild perturbations of G2 and M time [36], [37]. Cyclin B1 depletion produces a delay in mitotic entry and transit, and we reported that cyclin B2 depletion reduces the fraction of mitotic cells [37]. Thus, cyclin B2 appears to redundantly permit mitosis in cells that express less than 5% of peak Cyclin B1 levels in G2 [37]. These data fit models whereby cyclin B2 can either compensate for cyclin B1 loss or cyclin B2 can catalyze effective use of low, insufficient levels of cyclin B1. To explore the differential effects of these B cyclins on mitotic time, we examined quantitatively the effect of cyclin B1 or cyclin B2 expression on mitotic time and the ability of either cyclin to overcome the G2 arrest imposed by depletion of both B cyclins. To do this, we generated stable HeLa cell lines with inducible expression of cyclin B1-EGFP or cyclin B2–EGFP recombinant genes that are not recognized by cyclin B1 and B2 siRNAs.

## Materials and Methods

### List of stable cell lines used for experiments

Cells were single-cell sorted by FACS for B cyclin-EGFP or EGFP expression into 96 well plates. The following clones were used in this study: p001-1, (expresses EGFP); p006-14, p015-1, p015-4, p015-14 and p016-4 (express cyclin B1-EGFP); p010-5, p017-4, and p023-1 (express cyclin B2-EGFP). p015-1, p015-4, p015-14 and p017-4 expressed fusion RNA encoding 4 synonymous mutations in the siRNA recognition site, and p016-4 and p023-1 expressed fusion RNA encoding 7 and 6 synonymous mutations, respectively. p006-14 and p010-5 expressed fusion RNA with wild-type sequences. Since the same clones are used repeatedly throughout the study, the clone names have been abbreviated leaving off the clone number in the text and figures. Clone numbers are in Figure legends.

### Generation of stable cell lines, cell culture and transfection

HeLa BD™ Tet-Off cells (Tet-Off), and plasmids pTRE2hyg and pd4EGFP-N1 were purchased from Clontech (#630921, #6255-1, and #6072-1). Human cyclin B1 and cyclin B2 cDNA were amplified by polymerase chain reaction (PCR) using the following primers: CCNB1Nhe1-5′ - GGGGCTAGCGCCGCCACCATGGCGCTCCGAG, CCNB1Age1-3′ - CCCACCGGTGCCACCTTTGCCACAGC, CCNB2Nhe1-5′ - GGGGCTAGCGCCGCCACCATGGCGCTGCTCCG, CCNB2Agel-3′ - GGGACCGGTGCGGACCTTCCTATCAGTG, plasmids pcDNA3-cyclin B1 (a gift from Dr. Dennis Templeton, University of Virginia) and pcDNA3-cyclin B2 (a gift from Dr. Joelle Sobczak-Thepot, University P & M Curie). The EGFP cDNA was obtained by double digestion from the pd4EGFP vector, which codes for a mutant EGFP with a half-life reduced to 4 h. The cyclin B1 or cyclin B2 cDNAs with EGFP cDNA, or EGFP cDNA alone were cloned into the multiple cloning sites (MCS) of pTRE2hyg vector to generate pTRE2-cyclin B1-EGFP and pTRE2- cyclin B2-EGFP and pTRE2-EGFP constructs. These plasmids coded for fusion proteins with carboxy terminal EGFP. To generate cyclin B1 and cyclin B2 mutants that cannot be recognized by cyclin B1 and cyclin B2 specific siRNAs, 4 and 7 nucleotides in cyclin B1 siRNA recognition sites were mutated and 4 and 6 nucleotides in cyclin B2 siRNA recognition sites were mutated by PCR-mediated site-directed mutagenesis using the QuickChange kit (Stratagene, Catalog #200513). The mutants conserve the amino-acid sequence of cyclin B1 or cyclin B2. These constructs were named by pTRE2-m4cyclin B1-EGFP, pTRE2-m7cyclin B1-EGFP, pTRE2-m4cyclin B2-EGFP, and pTRE2-m6cyclin B2-EGFP respectively. To establish the stable cell lines described in Table 1, all the constructs were transfected into HeLa Tet-Off cells. EGFP expressing cells were sorted by FACS into 96-well plates and cultured in the presence of 200 ug/ml hygromycin B (Clontech, catalog# 631309) and 100 ug/ml G418 (Clontech, catalog #631307).

**Table 1:** Synonymous base changes.

Cell Line	Encoded genes	Sequences
p001	EGFP	
P006	Cyclin B1-EGFP	AAA CTT TCG CCT GAG CCT ATT
p015	Cyclin B1-EGFP	AAA CT**C** TC**C** CCT GA**A** CC**G** ATT
p016	Cyclin B1-EGFP	AA**G** CT**C** TC**C** CC**G** GA**A** CC**G** AT**T**
p010	Cyclin B2-EGFP	CAG CAC ACT TTA GCC AAG T
p017	Cyclin B2-EGFP	CAG CA**T** AC**C** TTA GC**G** AA**A** T
p023	Cyclin B2-EGFP	CA**A** CA**T** AC**C** TT**G** GC**G** AA**A** T

HeLa Tet-Off cells and established stable, clonal cell lines were plated in 6 or 10 cm tissue culture plates, maintained in RPMI (Invitrogen, catalog# 11835-055) supplemented with 10% fetal bovine serum (FBS, Gibco/Life Technologies, catalog #631101), 50 ug/ml gentamicin (Sigma, catalog# G1264-5G), 100 ug/ml G418, and 5 ng/ml Doxycycline (Sigma, catalog# D9891) at 37°C in a humidified atmosphere containing 5% CO_2_.

The target sequences against cyclin B1 and cyclin B2 have been previously described [37]. Briefly, oligonucleotides targeting cyclin B1 and cyclin B2 corresponding to the cDNA sequences of human cyclin B1 coding region 340–360 and human cyclin B2 coding region 845–855 were designed as recommended [38] and synthesized by Thermo Scientific. For siRNA transfection, cells were plated in 6 cm plates, and Lipofectamine 2000™ (Invitrogen, catalog #11668-019) was used according to the manufacturers' protocols.

### Cell fixation, intracellular staining and flow cytometry

Cells were fixed with 0.125% formaldehyde in culture media at 37°C for 10 min, washed, then followed by ice-cold methanol (90% final) as previously described [39]. For indirect staining, 1-2 million cells were incubated with antibody for 1 h at 4°C, washed twice with PBS/BSA (20 mg/ml bovine serum albumin, 10 mM NaPO_4_, 150 mM NaCl), followed by the secondary antibody for another 1 h at 4°C, and a final three washes. Finally, the cells were stained with 1 ug 4′,6-diamidino-2-phenylindole (DAPI) for DNA staining and analyzed by flow cytometry. For direct staining, the second antibody step was omitted. The following antibodies and amounts were used for cell staining: cyclin B1, 0.125 ug GNS1 conjugated with Alexa Fluor 647 (A647) or 0.125 ug GNS1 unconjugated (clone GNS1, BD Biosciences, catalog #554177); cyclin B2, 0.34 ug antibody (clone H105, catalog #sc-22776, Santa Cruz Biotechnologies), phospho-S10-histone H3 (PHH3), 0.125 ug conjugated with Alexa Fluor 488 (A488) (catalog #9708) or A647 (catalog #9716, Millipore-Upstate); phospho-T56-Bcl2, 0.25 ug (catalog #2875, Cell Signaling Technology); 5-bromo-2′-deoxyuridine (BrdU), 0.5 ug conjugated with A647 (clone PRB-1, Phoenix Flow Systems); Secondary antibodies with labels A488, Alexa Fluor 555 (A555), phycoerythrin (PE), and A647 were obtained from Invitrogen and used at 2:1 ratio w/w and 1 ug DAPI (Invitrogen). Stainings were performed in 50 ul phosphate buffered saline with 2% bovine serum albumin (BSA, Sigma, catalog #A7030-100G).

Multi-color fluorescence emmisions were measured using a BD Biosystems LSR II or Beckman Coulter (Miami, FL) Gallios. Two color measurements of EGFP and DNA were made on an Beckman Coulter XL. Cell sorting was performed with a BD Biosystems FACS Aria.

### BrdU labeling, G2 and M phase transit time analysis

Cells were continuously labeled with 20 uM BrdU (Sigma, catalog B9285) for various periods of time. At specific times, cells were harvested and fixed for Brdu staining as previously described [37]. Unlabeled G2 and M phase cells were quantified as a percentage of the population by Boolean classification and histogram analysis. G2 and M phase transit times were calculated as described previously [37] except that data were normalized to a top of 1 and bottom of 0 and analyzed by non-linear regression using decay equations following the form
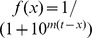
where *x* = the fraction of unlabeled G2 or M cells and t = time in hours.

### Time lapse experiments

Live cell images were obtained with a Leica AF6000 fluorescence microscope. Cells were cultured in glass-bottom dishes (ibidi, #81156) were placed into a covered sample chamber supplied with 5% CO_2_ and 37°C for 30 min before acquiring images, which were collected with a Hamamatsu C 10600-10B (ORCA-R2) camera through an HCX PL FLUOTAR L 20×0.40 DRY objective lens. Images were captured with 100–800 ms exposure times in 3 or 5 minute intervals for 18–36 hours.

### Immunoblotting

Cells were lysed in whole cell lysis buffer (0.137 M saline, 2% NP-40, 20 mM Tris, 1% sodium dodecylsulfate (SDS), 1% deoxycholate). 10–15 ug total protein was subjected to 8 or 10% SDS-polyacrylamide gel electrophoresis (PAGE) and transferred to polyvinylidene fluoride (PVDF) membrane (EDM, Millipore, catalog #IPVH09120). The following primary antibodies were used according to the manufacturer's instructions. Mouse anti-cyclin B1; Rabbit anti-cyclin B2; Goat anti-cyclin B2 (N-20, Santa Cruz, catalog #sc 5235); Rabbit anti-human cyclin A (upstate biotechnology, catalog #06-138); Mouse anti-Cdk1/Cdc2 (BD Transduction Laboratories, catalog #610038), Mouse anti-GFP (Santa Cruz, catalog #SC 9996) and Rabbit anti-β-tubulin (Abcam, catalog #ab6046-200). Alkaline phosphatase conjugated secondary antibodies were purchased from Promega. Membranes were developed by CDP-star chemiluminescent substrates (Sigma, catalog #C0712-100 ML). Kodak X-OMAT AR films (Eastman Kodak company, catalog #894 1114) were used to detect chemiluminescence.

### Software

Multi-parametric cytometry fluorescence data were analyzed using WinList 3D 7.0 software (Verity Software House). Cell cycle analysis was performed with ModFit LT 3.1 (Verity Software House) using single trapezoid S phase components and floating G1 and G2+M means and standard deviations. Resulting statistics and some secondary calculations were compiled and performed in Excel 2007 (Microsoft Corporation). Kinetic data were transformed and analyzed by non-linear regression in GraphPad Prism 5.04 (GraphPad Software). Prism was also used to construct x-y plots for Figures. Bivariate histograms were configured with WinList 3D and complex Figures composed with Adobe Photoshop 11.0.2 (Adobe Systems, Inc). Time lapse images were processed using Leica AF6000 imaging software.

## Results

### Stable cell lines express siRNA resistant B cyclin-EGFP proteins

**Figure 1:**
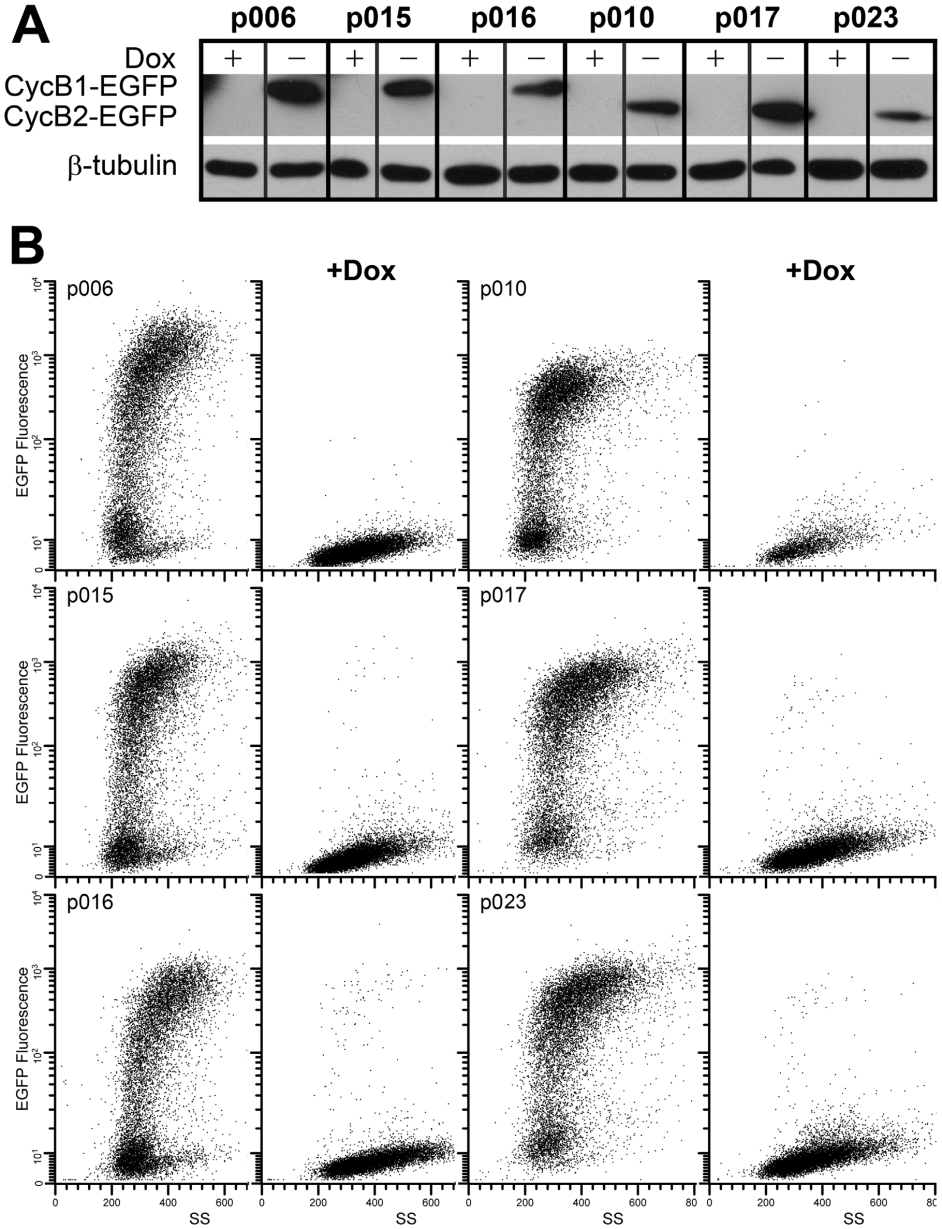
Expression of cyclin B1- and cyclin B2-EGFP is inducible and tightly regulated by Dox. HeLa cell clones p006-14, p015-4, p016-4, p010-5, p017-4, and p023-1 were cultured without and with 10 ng/ml Dox for 24 hr. **A:** Immunoblots with anti-GFP or anti-β-tubulin (loading control) antibodies. **B:** Cultured cells were fixed and subjected to the flow cytometry to measure the EGFP fluorescence. Bivariate histograms show EGFP fluorescence versus side scatter (SS) of indicated cell lines without and with Dox.

**Figure 2:**
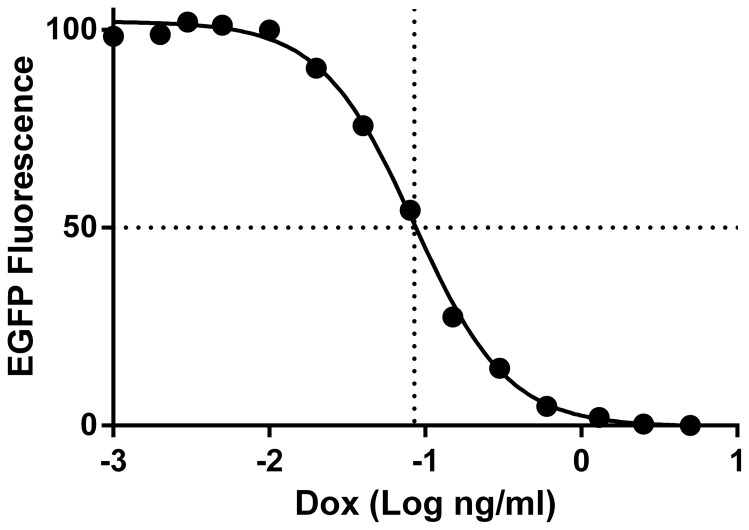
CyclinB1-EGFP expression is dose dependent. p006-14 cells were incubated with Dox at final concentrations from 0–10 ng/ml for 24 hr, then subjected to flow cytometry to measure EGFP fluorescence. The mean values for EGFP fluorescence for all cells were calculated then normalized to a scale of 0 to 100. Several other cyclin B1-EGFP and cyclin B2-EGFP lines were tested with similar results.

To study the effect of cyclin B1 and cyclin B2 on mitotic time and G2 arrest, we engineered stable HeLa cells to conditionally express human cyclins B1 and B2 from plasmids encoding sequences as shown in **Table 1**. The variant, synonymous sequences included 4, 6, or 7 mutations that rendered expression resistant to siRNA knockdown. The cyclins were expressed as C terminal tagged EGFP fusion genes, as previously reported. Immunoblotting and flow cytometry showed that ectopic EGFP or B cyclin-EGFP proteins of the stable cell lines were expressed or repressed in the absence or presence of Doxycycline (Dox), respectively (**Figure 1**). Time course experiments showed that expression of ectopic B cyclin-EGFP was maximal and stable by 13 hours after removal of Dox and minimal and stable by 17 hours after addition of 10 ng/ml of Dox (data not shown). **Figure 2** shows the dose response for EGFP expression in p006 and p010 cells. The IC50s were 0.04 (p006) and 0.07 (p010) ng/ml.

**Figure 3:**
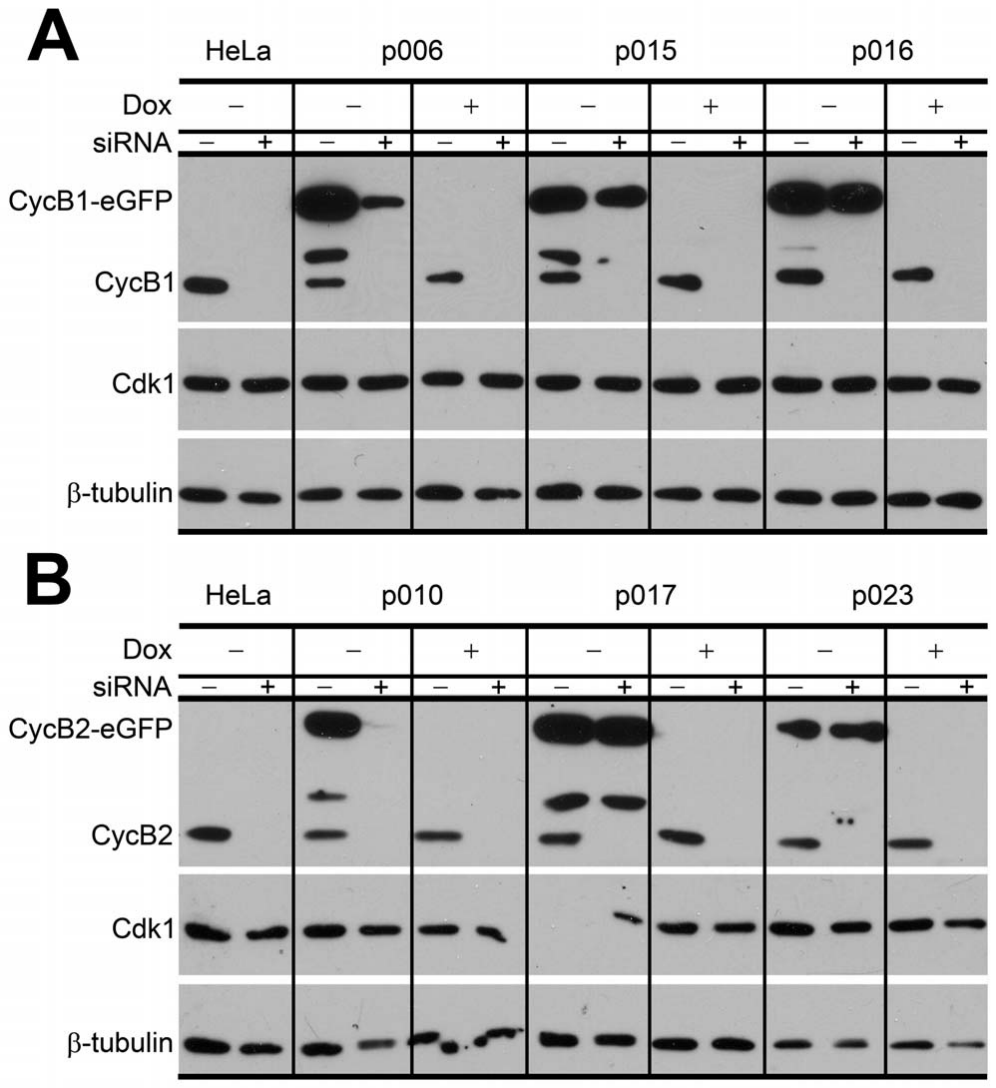
Expression of endogenous and exogenous cyclins as a function of Dox and cyclin B. **A:** p006-14, p015-4, p016-4 cells and HeLa Tet-Off control cells were transfected with cyclin B1 or control siRNA in the absence or presence of Dox for 18 hr. Cell lysates were immunoblotted with anti- cyclin B1, Cdk1, and β-tubulin antibodies to detect endogenous cyclin B1 and ectopic cyclin B1-EGFP. **B:** p010-5, p017-4, p023-1 and HeLa Tet-Off control cells were treated as above, except siRNA and antibody were specific for cyclin B2.

To examine the resistance of ectopic B cyclin-EGFP to siRNA knockdown, the stable cell lines were transfected with cyclin B1 or cyclin B2 siRNA for 18 h, followed by immunoblotting. By immunoblot, after siRNA treatment, endogenous B cyclins were undetectable; ectopic B cyclin-EGFP levels were unaffected (**Figure 3**: p015, p016, p017, p023), and exogenous cyclins with wild-type sequences were efficiently reduced (p006, p010). Dox treatment reduced exogenous proteins below the level of detection. Cdk1 levels were unaffected by any treatment. Therefore, all data showed that induction of ectopic B cyclins was efficient and tightly regulated by Dox, and exogenous cyclins with synonymous mutations were resistant to the B cyclin specific siRNA.

The B cyclin-EGFP proteins were localized appropriately within the cell lines. Cyclin B1-EGFP could be detected in the cytoplasm and on centrosomes during interphase and in the nucleus in prophase cells. Cyclin B2-EGFP was cytoplasmic, and co-localized with centrosomes and the Golgi during interphase. Cyclin B2-EGFP also entered the nucleus during mitosis (data not shown, see discussion).

### Ectopic cyclin B1 or cyclin B2-EGFP mediate Bcl-2 phosphorylation at threonine 56

**Figure 4:**
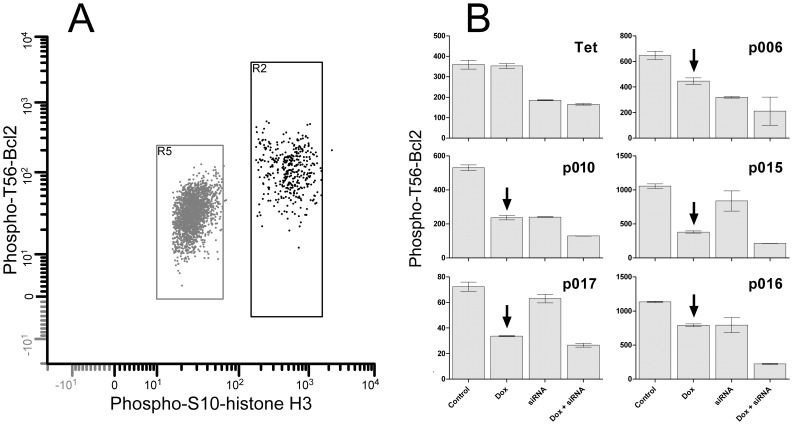
Bcl2 is phosphorylated as a function of cyclin B1- and B2-EGFP expression. Cyclin B1-EGFP clones p006-14, p015-4, p016-4, and cyclin B2-EGFP clones p010-5, p017-4, and HeLa-Tet-Off cells (Tet) were treated with control or cyclin B1/B2 specific siRNA in the presence or absence of 5 ng/ml Doxycycline for 24 hr. Cells were fixed, stained immunofluorescently for phospho-S10-histone H3 (PHH3) and phospho-T56-Bcl2, then analyzed by flow cytometry. **A:** expression of the two phospho epitopes by cytometry; R2 = mitotic cells; R5 = G1 cells. **B:** Mean phospho-T56-Bcl2 fluorescence of mitotic cells in the indicated cell lines when cells express cyclin B1- or B2-EGFP and endogenous cyclins (Control), express only endogenous cyclin B1 or B2 (Dox), express only ectopic cyclin B1- or B2-EGFP (siRNA), or express neither endogenous nor exogenous B cyclins (Dox+siRNA). Control and Dox values for Tet cells are equivalent because these control cells don't express B cyclin-EGFP. Arrows point to reduction in phospho-Bcl2 by addition of Dox.

Bcl2 is a substrate of Cdk1 [40]. To determine whether exogenous B cyclins were active, we measured the levels of phospho-T56-Bcl2 (pBcl2) by flow cytometry in mitotic cells cultured in the presence and absence of Dox and/or cyclin specific siRNA. The results are presented in **Figure 4**. The levels of pBcl2 in mitotic cells of the parent cell line (Tet) were unaffected by Dox treatment, but the levels for cell lines expressing cyclin B1-EGFP (p006, p015, p016) or cyclin B2-EGFP (p010, p017) were markedly reduced by Dox (arrows). Specific siRNA treatment reduced pBcl2 levels by variable amounts, and siRNA + Dox reduced levels maximally, except in the parent Tet-Off cells. The cells that were measured in the siRNA or siRNA+Dox samples included mitotic cells that did not knockdown. These data demonstrate that the activity of the EGFP fusion proteins was significant and equivalent to or greater than endogenous proteins.

### Cyclin B2 is rate controlling for G2 and M transitions

**Figure 5:**
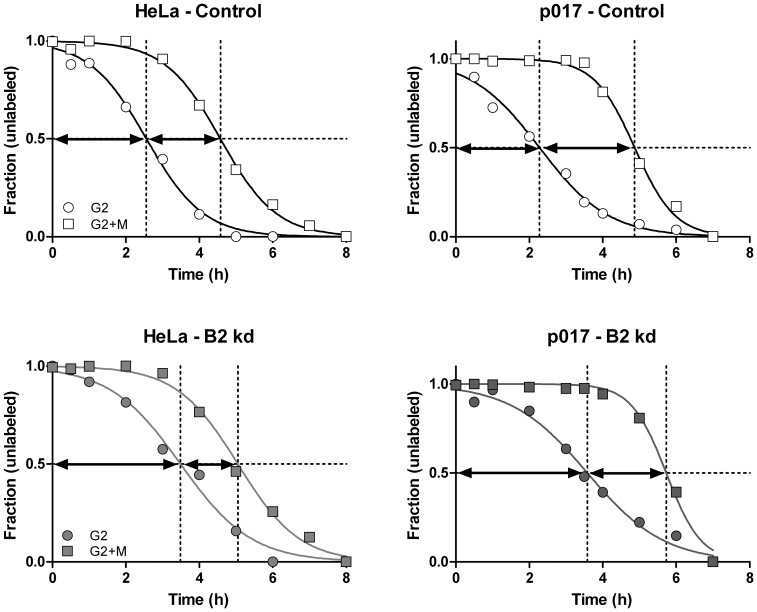
G2 and M phase transitions after cyclin B2 knockdown. HeLa or Dox treated p017-4 cells were transfected with control or cyclin B2 siRNA (B2 kd) for 18 hours, then continuously labeled with BrdU. Cells were then trypsinized, fixed, and immuno-stained for BrdU, PHH3, (and cyclin A2 in one experiment) and DNA content, then measured by flow cytometry. The frequencies of unlabeled G2 and M cells were quantified as previously described [37] and plotted versus time. The distance between the origin and the t_1/2_ point of the G2 curve (circles) = the average G2 time and the difference between the t_1/2_ of the M (squares) and G2 curves = the average M phase time. The values calculated from these data are presented in Table 2 (p017 and HeLa).

We have shown previously that depleting the levels of cyclin B1 produces an increased fraction of G2 and M phase cells, and that these increased frequencies result from delays in G2 and M transitions. Further, we reported that cyclin B2 knockdown increases the fraction of G2 phase but decreases the M phase fraction. This suggested rate limiting functions for cyclin B2, but the effects were small [37]. Further, the reduced M phase fraction could indicate that the M transit rate was either unchanged or increased by cyclin B2. This might make sense if a function of cyclin B2 is to regulate Golgi fragmentation, provided that Golgi fragmentation and dispersal is necessary, and that there is a checkpoint function involved [41]. To explore the rate limiting nature of cyclin B2, we performed kinetic experiments to determine G2 and M phase times for cells with depleted cyclin B2. **Figure 5** shows typical kinetic data and **Table 2** shows the results of several experiments with two B2-EGFP cell lines and with a HeLa cell lab strain that is distantly related to the parental Tet-Off cell line. In all experiments, cyclin B1 depletion always resulted in lengthened G2 and M phases (data not shown). Most of the time, cyclin B2 depletion resulted in a longer G2 but shorter M, supporting our original observations [37]. In some experiments where cells were growing slower (t_G2_>3 h), cyclin B2 depletion resulted in longer M phase times, therefore, the shortening of M by cyclin B2 depletion didn't always occur, or there were experimental problems that we couldn't define. To investigate this further, we did a series of time-lapse analyses. Average data for 7 experiments showed an average mitotic time of 81 and 50 minutes for control and cyclin B2 knockdown cells, respectively (N = 34; N = 38). The results were significant (p<0.0001) using a Mann Whitney test, since the values were not normally distributed for knockdown cells. These experiments were done without a nuclear marker, and we did not track cell density. We performed four more experiments after transfection of histone H2b-GFP to mark the chromatin. Each experiment showed significant differences after cyclin B2 knockdown with an average reduction in mitotic time of 23% (range = 19–28%; p = 0.025, 0.0009, <0.0001, <0.0001); N = 12 and 15; 8 and 10, 45 and 48, and 20 and 11 for controls and knockdown, respectively). This last series was done at three different cell densities. The forgoing provide two lines of evidence for rate limiting roles for both B cyclins and opposing effects on M phase time under conditions of siRNA knockdown of the opposite cyclin.

**Table 2:** G2 and M phase times; effect of cyclin B2 knockdown.

			Controls		Effect of Kd	
Cyclin Kd	Cell Line	Experiment	t_G2_ (h)	t_M_ (h)	Δt_G2_ (h)	Δt_M_ (h)
	p006	1	2.4	2.0		
	p006	2	1.8	1.6		
	p015	1	2.2	2.1		
	p016	1	2.0	1.9		
	p010	1	2.1	2.4		
B2	p010	2	1.7	2.4	1.3	−0.2
	p017	1	1.5	2.9		
B2	p017	2	2.3	2.6	1.3	−0.4
B2	HeLa	1	2.5	2.2	1.1	−0.3
B2	HeLa	2	2.6	1.8	0.9	−0.5
**Means**			2.1	2.2	1.1	−0.37

Δt_G2_ = t_G2_ (B2 Kd)−t_G2_ (Control); ΔM_G2_ = M_G2_ (B2 Kd)−M_G2_ (Control). Cell line controls were treated with Dox to eliminate expression of exogenous B cyclin-EGFP. Rows with blank cells represent experiments without knockdown treatment and are included to better describe averages for t_G2_ and t_M_.

### Over-expression of B cyclins does not affect the rates of G2 and M transition

**Figure 6:**
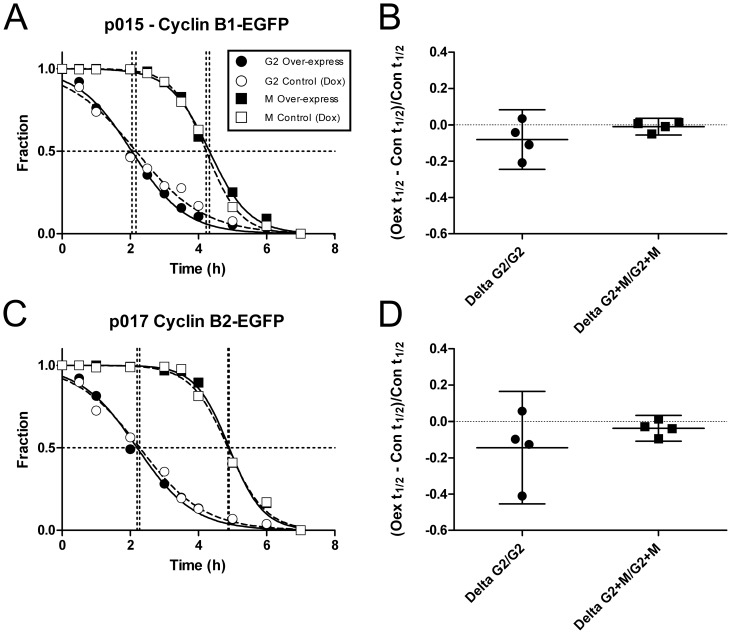
Over-expression of B cyclins does not alter G2 and M phase transition times. P006-14, p015-4 and p010-1, p017-4 cells were cultured with BrdU in the presence (Control) or absence of Dox (Over-express) to test the ability of B cyclin over-expression to decrease G2 transit and affect M transit. Cells were fixed, stained, and measured as in Figure 5. **A**, **C:** fractions of unlabeled G2 (circles) or M (squares) cells as a function of time in the absence (white symbols) or presence of Dox (black symbols). **B**, **D:** two experiments with p006-14, p015-4, p010-1, and p017-4 were analyzed as in A, C and the transit times calculated. To normalize the data the differences between Dox treated and untreated G2 and G2+M t_1/2_ values (dotted lines) were divided by the t_1/2_ for Control cells (Y axis). The data for G2 (Delta G2/G) and G2+M (Delta G2+M/G2+M) are denoted on the X axis. The results are not statistically different from 0 for any data set.

Unless the endogenous B cyclins are expressed at saturating conditions for their ability to affect cell cycle time, we expected that increasing the B cyclin concentrations would result in increased transition rates through G2 for both cyclins and also through M for cyclin B1. Presumably, (under the simplest model) expressing cyclin B2 at higher levels would decrease the M phase transition relative to controls. To address this, we performed kinetic experiments to examine effects of over-expression of B cyclin-EGFP on G2 and M times in the conditional expressing cell lines. We compared the rates of transition in two cell lines for each cyclin and two experiments for each line. The cells were cultured with and without Dox, providing either endogenous cyclin levels (Dox) or over-expression of B cyclin-EGFP in excess of endogenous B cyclins. **Figure 6A, C** shows typical results and **Figure 6B and D** show the summary of experiments. In both cases, the transition rates in the presence of exogenous B cyclin were not significantly different from the rates in the presence of Dox, although the majority of the samples registered a slight increase in transition rates. This indicates that endogenous cyclins are expressed at "saturating" levels for the ability to control G2 and M transition times under exponential growth conditions. This could be considered tight regulation of B cyclin activities under "wild-type" conditions.

### Over-expression of single B cyclins can restore cell cycling when endogenous B cyclins are depleted

**Figure 7:**
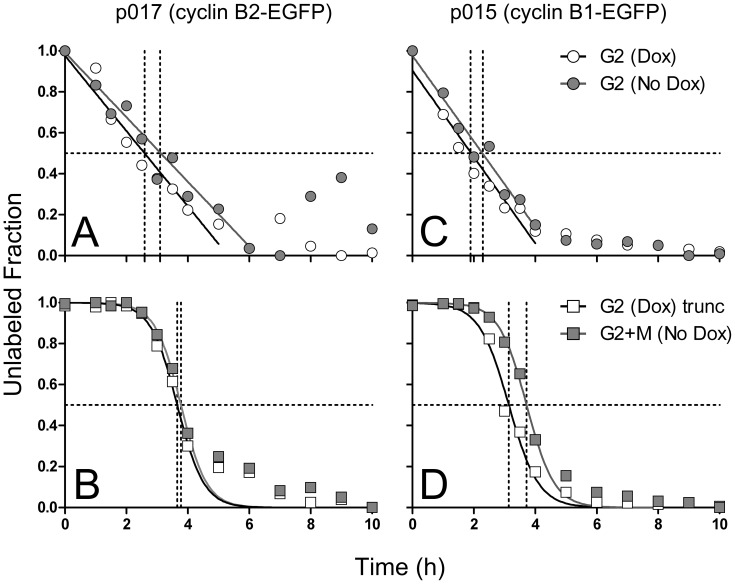
B cyclin-EGFP expression rescues the G2 arrest phenotype of B cyclin co-depletion. Cyclin B2-EGFP conditional expressing p017-4 cells (A,B) and cyclin B1 p015-4 cells (C,D) were transfected with control siRNA and cultured in the presence of Dox (white symbols) or transfected with both B cyclin siRNAs and cultured without Dox (gray symbols). Cells were continuously labeled with BrdU, and at timed intervals, fixed and stained for cell cycle analysis. **A**, **C:** Decay curves of the unlabeled G2 fractions. **B**, **D:** Decay curves of the unlabeled G2+M fraction. The experiment was performed in duplicate; the mean values are plotted. Regressions were performed on samples from time 0–4 hours.

Double knockdown of both B cyclins results in a profound G2 arrest followed by 4C cell cycles [36], [37]. This profound arrest led us to conclude that either B cyclin could promote mitotic transition, since the arrest argued that any residual level of cyclin B1 in single-cyclin B1 depletion experiments was insufficient to catalyze mitotic transit unless normal levels of cyclin B2 were present. Therefore, with these conditional expression cell lines, we could ask whether a single cyclin could completely rescue cells from the profound G2 arrest and promote mitotic transition. B1 and B2-EGFP cell lines were treated with either control siRNA and Dox (expressing endogenous, normal levels of B cyclins) or B cyclin specific siRNA without Dox (over-expressing specific B cyclin-EGFPs). Eighteen hours after transfection, cells were continuously labeled with BrdU over a period of 10 hours, sampled and subjected to kinetic analysis. The results, shown in **Figure 7**, demonstrate that either B cyclin-EGFP could rescue cells from the effects of double siRNA mediated B cyclin depletion. For both cyclins, the rescue was almost to the transition rates of controls for both G2 and M.

### Expression of single B cyclins can completely rescue the G2 arrest phenotype caused by cyclin B1 and B2 co-depletion

**Figure 8:**
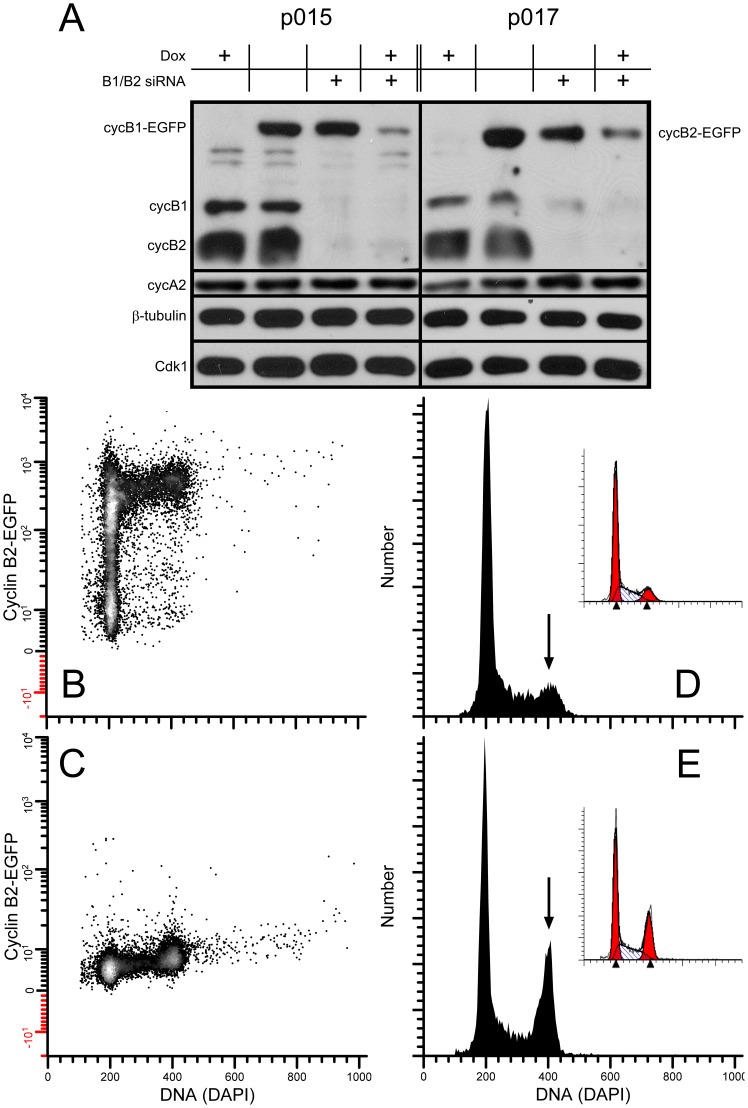
B cyclin co-depletion induces G2 arrest. HeLa-p015-4 and HeLa-p017-4 cells were transfected with control siRNA or B cyclin siRNAs in the presence or absence of Dox for 24–30 hours. Cell lysates were then subjected to electrophoresis and immunobloting (**A**) or fixed and stained for flow cytometry (**B**). **A:** Immunoblot analysis of cyclin B1 (cycB1), cyclin B2 (cycB2), cyclin A2 (cycA2), Cdk1, and β-tubulin under noted conditions. **B**, **C:** example cyclin B1-EGFP expression for p015 in the absence (B) or presence (C) of Dox. **D**, **E:** DNA content histograms of control cells without Dox (D) or co-depleted cells with Dox (E). Arrows point to 4C cells. Cells depicted in E are both co-depleted for endogenous cyclins and repressed (Dox treated) for exogenous cyclins.

**Figure 9:**
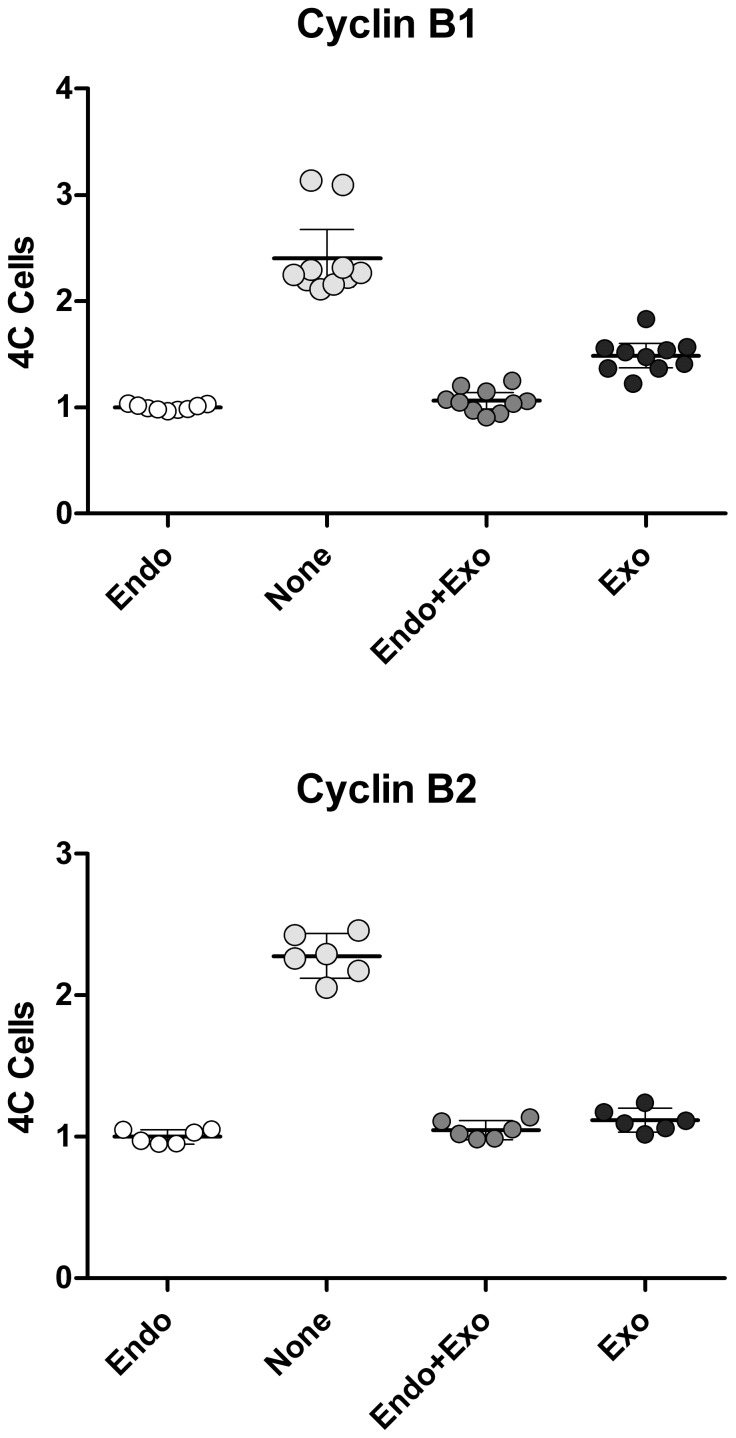
Single B cyclin-EGFP can rescue the G2 arrest of endogenous cyclin B1 and B2 co-depletion. Two clonal cell lines for each B cyclin and two independent experiments were performed as in Figure 8. The percentage of 4C cells in control cells (Endo) were normalized to 1. Co-depletion increases G2 cells by 2 to 3 times (None). Expression of B cyclin-EGFP in the presence of endogenous cyclins did not change G2 fractions (Endo+Exo). Expression of single B cyclin-EGFP in the presence of endogenous cyclin co-depletion restored G2 fractions close to normal cycling levels (Exo).

**Figure 10:**
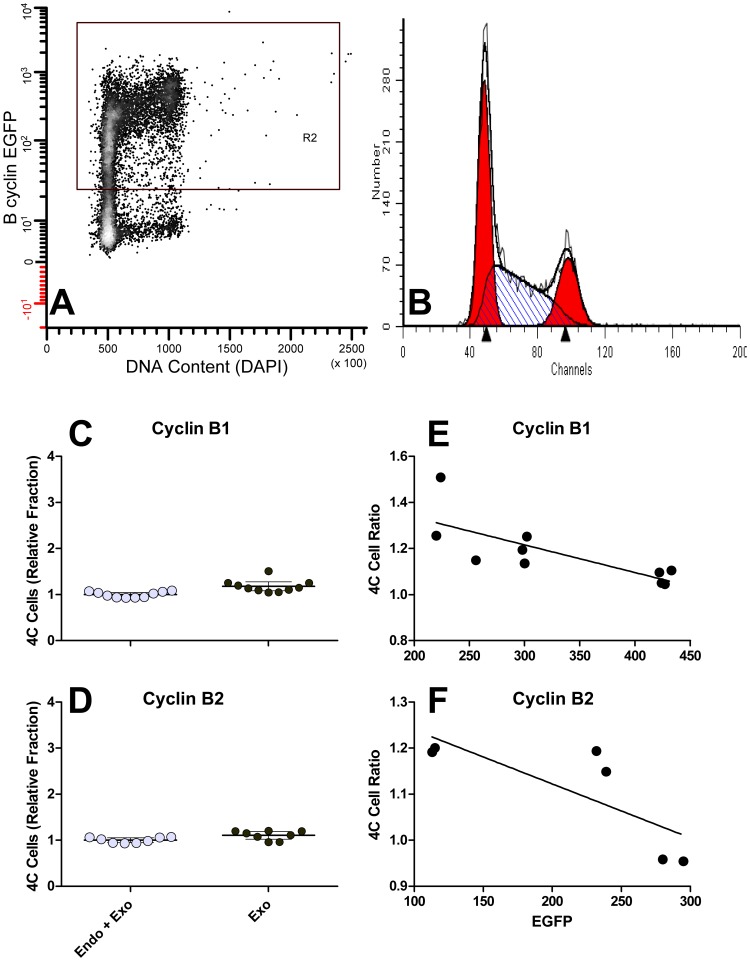
Reanalysis of the primary data of the experiments presented in Figure 9. Each data file was gated (R2) as shown in **A**. Cell cycle analysis was performed as in **B**. The 4C fractions were plotted for the p015 (**C**) and p017 (**D**) cells for the treatments that resulted in expression of B cyclin-EGFPs. Rescue of the knockdown phenotype is shown in **E** and **F** as a function of EGFP expression levels. The slope for the data in E is significant (p = 0.01); the slope for the data in F is not (p = 0.06). The 4C cell ratio is the ratio of the 4C cells in co-depleted cultures (Endo + Exo: B specific siRNA without Dox) to those in rescued cultures (Exo: control siRNA without Dox).

The experiments presented above and in **Figure 6** require many samples and are demanding. To further examine the ability of a single B cyclin to overcome cell cycle arrest induced by knockdown of both B cyclins, we measured the fraction of 4C cells by DNA content flow cytometry after siRNA knockdown and Dox treatment. Cell lines, p015 and p017, were transfected with B cyclin specific siRNAs in the presence or absence of Dox for 24–30 hours, and the levels of endogenous B cyclin co-depletion was measured by immunoblotting (**Figure 8A**). Compared to control siRNA, endogenous B cyclins were barely detectable and cyclin A2 and Cdk1 levels were invariant. The reduction of cyclin B1, measured by flow cytometry as previously reported [37] was 99% of peak levels, and 67% of 4C cells displayed cyclin B1 immunofluorescence levels equal to that of late mitotic cells. Cyclin B2 knockdown is more difficult to determine, but our calculations based on immunofluorescence suggest that it approaches 100% for 80% of the 4C cells. **Figure 8E** shows the G2 arrest induced by siRNA treatment + Dox. **Figure 8D** shows the control DNA content distribution when cells are not treated with siRNA. **Figure 8** (B, C) shows an example of EGFP expression for p017 cells cultured without (B) or with (C) Dox. **Figure 9** presents data for the two clonal cell lines and two experiments each. The fractions of 4C cells were measured by DNA content modeling as shown in **Figure 8** (D, E insets). When both endogenous cyclins were knocked down and cells were treated with high concentrations of Dox (**Figure 9** **, None**), the fractions of 4C cells were between 2 and 3 times the fractions in cells treated with Dox alone and expressing only endogenous B cyclins (**Figure 9** **, Endo**). When cells express both endogenous and exogenous B cyclins (**Figure 9** **, Endo+Exo**), the fractions of 4C cells were equivalent to cells expressing only endogenous cyclins - emphasizing the previous conclusion that exogenous cyclins do not further affect cell cycle timing in the presence of endogenous cyclins. This is the case for both B cyclins. Full expression of exogenous cyclin B1-EGFP did not fully rescue the G2 arrest phenotype in these experiments, whereas cyclin B2-EGFP did (**Figure 9** **, Exo**). However, we noticed that cells that rescued least well were cells that either expressed lower levels of exogenous B-cyclin EGFP or the cultures contained S and G2 cells that were still negative for EGFP after full induction (no Dox). An example of this type of clone is shown in **Figure 10A**. We then re-analyzed the primary data from the experiments of **Figure 9**, performing cell cycle analysis on only the EGFP positive fraction (R2 gate in **Figure 10A**). This is presumably equivalent to cell cycle analysis on cycling cells after the loss of Cdh1 activity in G1. The comparable results are presented in **Figure 10C, D**. In **Figure 10E and F**, the ratios of 4C cells in the rescued cells to those in the knockdown are plotted versus EGFP expression levels. A ratio of 1 is equivalent to full rescue. The analyses presented in Figure 10 indicate that the co-depletion phenotype can be fully rescued when B cyclin EGFP expression is high enough. All of the analysis in Figure 10 takes advantage of the fact that expression of exogenous B cyclin EGFP in the presence of endogenous cyclins does not alter cell cycle time (**Figures 6**, **8**)

**Figure 11:**
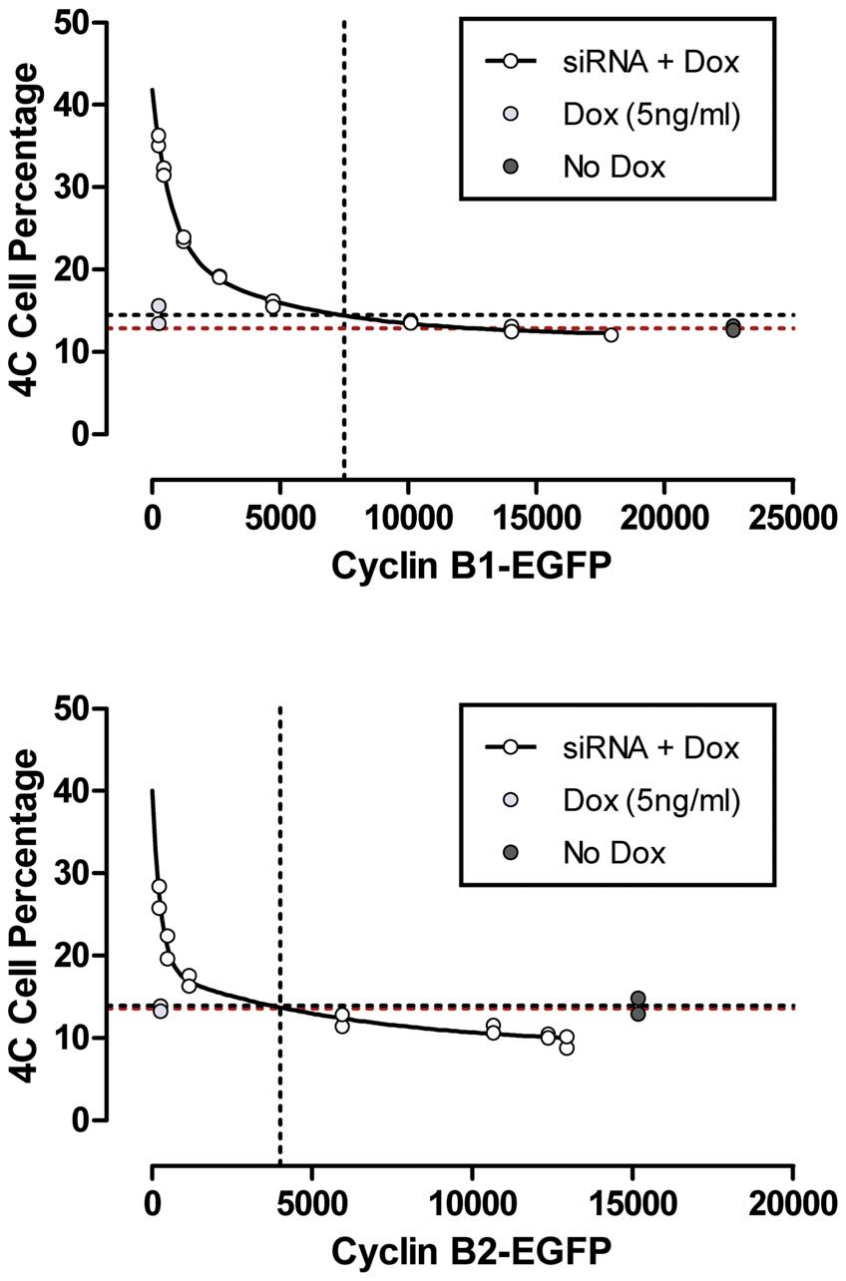
Single B cyclin EGFP completely rescues the G2 arrest of cyclin co-depletion as a function of dose. p015-4 and p017-4 cells were transfected with B cyclin siRNA in the presence of Dox at stepped levels for 24–30 h. EGFP fluorescence and the fraction of 4C cells were measured by flow cytometry (siRNA + Dox). Controls were transfected with scrambled siRNA and expressed endogenous and exogenous B cyclins (No Dox) or only endogenous cyclins (Dox).

Therefore, we used another p015 clone, selected for high expression (p015-1) and sorted the p017 clone by FACS to obtain a sub-line that fully expressed high levels of B2 cyclin EGFP. We then performed an experiment, measuring the 4C fraction of cells as a function of B cyclin EGFP expression dose. The results are presented in **Figure 11**. For cyclin B1, expression of B1-EGFP could fully compensate the phenotype, and for cyclin B2-EGFP, the fraction of 4C cells could be lowered significantly below the levels produced by both endogenous B cyclins. This experiment was repeated with similar results. The simplest explanation, especially in view of the data presented in **Figure 7** for reduction of 4C cells, is that cells do not arrest in G2 and continue through M to G1. This experiment also shows that in the double knockdown cells, full expression of either B cyclin-EGFP is less than full expression of B cyclin-EGFP in control cells with endogenous cyclins. We do not know the reason for this, but checking all related experiments, the finding was consistent with about 15% less expression when the endogenous cyclins were not present.

## Discussion

### The effects of B cyclin knockdown in cell lines are mild

We were surprised when we first observed the results of siRNA knockdown of cyclin B1 [37]. We expected that cells that had not entered M would arrest in G2, and that cells in mitosis would prematurely exit mitosis, since it was known that high levels of cyclin B1/Cdk1 activity prevented mitotic exit [42]. We expected that we would see a high fraction of G2 phase cells and reduced or approximately zero M cells. Instead, we showed that >95% knockdown of cyclin B1 resulted in ∼20% increases in the G2 and M transit times. An increased rate of endoreduplication was an associated cyclin B1 knockdown phenotype. We associated cyclin B1 knockdown with induction of high levels of maloriented chromosomes during metaphase (present only in HeLa cells) and sporadically increased cell death (present in both HeLa and RPE cells). In the same study, we also demonstrated that knockdown of cyclin B2 resulted in a slightly elevated G2 fraction and a reduced fraction of M cells. Finally, we and others [36] showed that double knockdown of cyclins B1 and B2 resulted in a profound G2 arrest that resolved eventually by transit to a 4C cell cycle. Bellanger et al. showed that this 4C phenotype could be reversed by transfection of cyclin B2 [36]. We have shown quantitatively that it could be completely reversed.

### Here, we focused on similarities and differences between cyclin B1 and B2 with respect to G2 and M transition rates

First, we completed the kinetic studies on cyclin B2 knockdown to provide more clarity to any differing cell cycle roles for the two cyclins, and second, we asked whether single cyclins could quantitatively compensate for the major effect of B cyclin co-depletion (G2 arrest). We found that the elevated fractions of G2 cells were due to longer G2 transit times, which is expected for a mitotic cyclin. We also found that the lower levels of M phase cells, previously noted upon cyclin B2 knockdown, resulted from shorter M transit times. On average, for a 2.2 hour M phase time, knockdown of cyclin B2 resulted in an M phase that was 22 minutes shorter, or a reduction of 17% (**Table 2**). We supported this with similar results from time lapse microscopy that showed an average 23% reduction. This is opposite of the effect of cyclin B1 knockdown. It was an expected outcome based on our previous frequency results, but in general somewhat surprising. The G2 lengthening was expected, but the magnitude is high. The average increase was 1.1 hour longer than the 2.1 hour average G2 time in control cells. This is approximately a 50% increase, which is the strongest B cyclin knockdown phenotype noted. Second, we asked whether over-expression of a single B cyclin could decrease transit times in the presence of endogenous cyclins. We could not find any evidence that either exogenous B cyclin was able to reduce G2 and M times in the presence of endogenous cyclins (**Figure 6**). Thus, although knockdown experiments indicate that the B cyclins are rate controlling, the expectation that these molecules could produce the opposite effects when over-expressed was not demonstrated. On another tack, we asked whether a single B cyclin could rescue the profound G2 arrest induced by knockdown of both endogenous B cyclins. The evidence from kinetic experiments is that either cyclin can rescue this phenotype (**Figure 7**). Expression of exogenous cyclin B2-EGFP nearly restored G2 transit time, but cyclin B2-EGFP could completely restore M transit, and in fact, reduce M transit times (**Figure 7**). This latter result is perplexing in that given the knockdown results, the simplest model would suggest that restoration of phenotype by B2 would lengthen M phase. However, the cyclin B2 knockdown experiments differ from the rescue experiments in that endogenous cyclin B1 is present in the single knockdown experiments. At this stage, the best explanation is that the system is more complex with respect to time than any simple model suggests. Expression of cyclin B1-EGFP in the absence of endogenous B cyclins nearly restored G2 and M transit times to those of endogenous cyclins (**Figure 7**). We supported these kinetic studies with experiments that monitored the fraction of 4C cells (G2+M) in cells that expressed only endogenous cyclins (treated with Dox), cells with siRNA knockdown of endogenous cyclins + Dox suppression of exogenous B cyclins (treated with B cyclin siRNAs and Dox), cells that expressed both endogenous and a single exogenous B cyclin (treated with control siRNA), and cells that expressed only a single exogenous B cyclin (treated with B cyclin siRNAs). These experiments reinforced the ability of either B cyclin to rescue the cell cycle arrest phenotype of double B cyclin knockdown (**Figures 8** and **9**). Finally, since we noticed that cells with partial expression or lower expression of exogenous cyclins appeared to rescue less well (**Figure 10**), we tested the ability of either B cyclin to restore the cell cycle arrest phenotype of B cyclin co-depletion by measuring the fractions of 4C cells as a function of single B cyclin-EGFP expression (Dox concentration). These results showed that when the expression of the exogenous B cyclin was high enough, cyclin B1 could completely restore the phenotype and cyclin B2 could exceed the phenotype - presumably by reducing G2 and M phase times (**Figure 11**). Thus, aside from the special phenotype of M transit shortening in the presence of endogenous cyclin B1, the two B cyclins appear completely redundant for the ability to regulate G2 and M phase transit times. Since the experiments of **Figure 11** were analyzed by cytometry on the same day and same instrument settings, the levels of EGFP expression are comparable, molecule for molecule. Curiously, cyclin B2 appears to be more potent for restoring the G2 arrest phenotype than cyclin B1. Cyclin B2 restores the levels of 4C cells to control levels at 4000 EGFP units while cyclin B1 requires 7800 units. This could be trivial in that cyclin B2-EGFP could be more active than cyclin B1-EGFP. Since cyclin B2-EGFP was able to reduce the 4C cell fraction below the level of cells expressing endogenous cyclins only or cells expressing endogenous plus exogenous cyclins at lower levels than the endogenous + exogenous expressing cells, the fact that timing is more complex than simple models suggest is emphasized.

### Implications for cell cycle models

There are three conceptual models for the general ability of cyclins to promote movement through the cell cycle. The first is a threshold model in which high affinity substrates can be phosphorylated at low cyclin concentrations and lower affinity substrates are phosphorylated at higher cyclin concentrations, which occur at a later point in time. There is significant evidence for this model [43]. The model works best to explain organisms like fission yeast with a single mitotic cyclin and a single Cdk, but evidence supports the model in more complex organisms [44], [45]. The second model is that specific cyclins direct Cdks to specific substrates. This model explains the periodic control by different cyclins and Cdks and is suited to organisms that display periodic expression of several cyclins and several Cdks (e.g., mammals) or one Cdk (budding yeast). There is also significant evidence for this model [46]. Finally, a third model considers that cyclin localization affects the ability to phosphorylate substrates. Thus, premature activation of nuclear targets by cyclin B1/Cdk1 are prevented (or, ensured) by dominant nuclear export until cyclin B1/Cdk1 activation at the beginning of mitosis [31]. These three models are not mutually exclusive and the most sensible, current model for mammalian cells is that all three concepts are in play [47].

Evidence for the role of localization is best illustrated by the B cyclins of mammals because localization is organelle-based and readily observed. Cyclin B1 is mainly cytoplasmic during interphase and abruptly accumulates in the nucleus during prophase, prior to nuclear envelope breakdown (NEB). In the cytoplasm, cyclin B1 is localized to microtubules and the centrosomes, and in the nucleus it is localized to chromatin and kinetochores. As mitosis progresses, cyclin B1 disappears from kinetochores first, then chromatin, and finally the spindle and centrosomes - the latter occurring at the same time as cyclin B1 degradation [48]. Cyclin B2 co-localizes with Golgi markers during interphase but is cytoplasmic after Golgi fragmentation, and disperses throughout the cells after NEB [31]. A small amount could be shown to localize with the spindle at metaphase when cells were fixed with acetone/methanol and stained immunofluorescently [24]. We have detected both the cyclin B2-EGFP and endogenous cyclin B2 in the nucleus during mitosis prior to nuclear membrane breakdown and co-localized with centrosomes and the spindle (data not shown).

Currently, the division of function for the two somatic B cyclins rests with Golgi fragmentation for cyclin B2 and nuclear mitotic functions for cyclin B1, including important roles in nuclear membrane breakdown, spindle assembly, and spindle checkpoint induction and maintenance [10]. Cyclin B1 shuttles between the cytoplasm and nucleus until activation of cyclin B1/Cdk1. Then, a dramatic change in nuclear import rate occurs, and cyclin B1 is retained in the nucleus [28], [49]–[51]. Peptide swapping experiments showed that a cyclin B1 fragment could be targeted to the Golgi by a cyclin B2 fragment, but cyclin B2 could not be targeted to the nucleus with an equivalent cyclin B1 fragment [31]. Equally, the localization of cyclin B1 to chromatin, kinetochores, the centrosome, and spindle could largely be attributed to specific peptide fragments [48].

### Alternative models

#### If cyclin B1 is unnecessary for mitosis in human somatic cells - Model (1)

Taking all of the forgoing into consideration, the simplest explanation for the high level of redundancy for G2 and M timing in B cyclin knockdown experiments is that in the absence of cyclin B2, cyclin B1 can bind to the Golgi and function well enough to regulate Golgi fragmentation. In the absence of cyclin B1, cyclin B2 functions well enough to compensate for cyclin B1, at least in short term tissue culture. This should mean that cyclin B2 binds to centrosomes, and either prior or after NEB, to chromatin, kinetochores, and the spindle. Jackman et al. first reported human cyclin B2 Golgi localization [24]. In that paper, centrosomal-like objects can be seen to be stained, but were not reported on. In addition, an image was presented that shows possible nuclear and cytoplasmic staining but was either interpreted to be diffuse cytoplasmic staining or was interpreted as evidence that complete re-location (like cyclin B1) was not occurring. Binding to the spindle was observed. Further studies from the same group focused on microinjected G1 cells to obtain localization and functional information [31]. The functional evidence presented is very convincing for different actions of the two cyclins and supports essential mitotic functions to cyclin B1 (aster formation and NEB). Since cyclin B1/Cdk1 has been observed to induce Golgi fragmentation "better" than cyclin B2 [31], the decrease in M phase transit time that we observe when cyclin B2 is knocked down could result from an ability of cyclin B1 to fragment the Golgi more efficiently (therefore faster) and satisfy a Golgi fragmentation checkpoint sooner [41] and thereby increase M transit rate (in absence of cyclin B2). This is speculative, since the putative Golgi checkpoint is only known to arrest cells in G2, and the rate limiting function we are concerned with should be in early mitosis [30]. We do not have evidence for increased localization of cyclin B2 to cyclin B1 structures; however, we have observed cyclin B2 immunostaining localized to the Golgi, centrosomes, the mitotic spindle, and in the nucleus in control and single knockdown experiments. We have also observed cyclin B2-EGFP localized to these same structures and observed nuclear entry at the beginning of mitosis in time lapse experiments. Thus, there are few arguments that cyclin B2 would not be in the right (larger) place to perform cyclin B1 functions (we have not detected it on kinetochores). However, the functional of study of Draviam [31] argues that cyclin B2 would be very inefficient regulating spindle assembly or NEB.

#### If cyclin B1 is necessary for mitosis in mammalian somatic cells - Model (2)

Recently, unpublished data was cited [46] to indicate that mouse cells genetically null for cyclin B1 arrest in G2. If this finding is supported, then a working model of full redundancy would be incorrect for mouse cells, and either a major difference between mouse and human somatic cells exists, or a different model is needed to explain all data. Assuming similarity between mouse and human, critical high affinity substrates that are phosphorylated at low levels of cyclin B1 (less than 3–5% of prophase levels) need to be postulated. The residual levels of cyclin B1 in double knockdown experiments would have to fall below that threshold, and for the simplest model, in single cyclin B1 knockdown experiments, the achieved knockdown levels would necessarily be above that critical threshold level. This possibility is the opposite of expected since double knockdown experiments are less efficient than single knockdown experiments, e.g. [37]. An attractive but more complex model is to postulate at least one critical substrate with primary and secondary B cyclin/Cdk1 phosphorylation sites. The primary site(s) would be low affinity and phosphorylated by high levels of either B cyclin/Cdk, and phosphorylation would be required prior to phosphorylation of the secondary site(s). The secondary site(s) would be a cyclin B1/Cdk1-specific, high affinity site(s) that could be phosphorylated by the low, residual levels of cyclin B1/Cdk1 after knockdown. Such substrates would explain the single and double knockdown data equally well and any data supporting a necessary role for cyclin B1. Because the high affinity site(s) would require low levels of cyclin B1, single cyclin B1 knockdown would result in continued cycling. The low affinity site(s) would be phosphorylated by high levels of cyclin B2/Cdk1 and the high affinity site(s) would be phosphorylated by residual cyclin B1/Cdk1. In double knockdown, neither cyclinB1 nor B2 would reach a level needed to phosphorylate the low affinity site(s), and cell cycle arrest would occur despite the presence of enough residual cyclin B1/Cdk1 to phosphorylate the secondary high affinity site(s).

The idea that the B cyclins are 100% redundant does not seem very likely given the long evolutionary time in which fitness should select for the most efficient system. Currently, our kinetic results suggest that there is a cost to the loss of cyclin B2 - less efficient transition into M and a faster rate through M. Both of these features might reduce to an ability to modify and optimize the putative Golgi fragmentation checkpoint [30], [41].
